# Evaluation of the difference in caries experience in diabetic and non-diabetic children—A case control study

**DOI:** 10.1371/journal.pone.0188451

**Published:** 2017-11-30

**Authors:** Stefano Lai, Maria Grazia Cagetti, Fabio Cocco, Dina Cossellu, Gianfranco Meloni, Guglielmo Campus, Peter Lingström

**Affiliations:** 1 Department of Biomedical Sciences, Medical School, University of Sassari, Sassari, Italy; 2 WHO, Collaborating Centre for Epidemiology and Preventive Dentistry, Milan, Italy; 3 Department of Biomedical, Surgical and Dental Sciences, University of Milan, Milan, Italy; 4 Department of Surgery, Microsurgery and Medical Sciences—Dental School, University of Sassari, Sassari, Italy; 5 Department of Surgery, Microsurgery and Medical Sciences—Clinic of Pediatric, University of Sassari, Sassari, Italy; 6 Department of Cariology, Institute of Odontology, Sahlgrenska Academy, University of Gothenburg, Gothenburg, Sweden; Forsyth Institute, UNITED STATES

## Abstract

**Aim:**

To evaluate the caries prevalence and related variables in Type 1 diabetic and non-diabetic children and among the diabetic children according to their metabolic status.

**Methods:**

Sixty-eight diabetic and 136 non-diabetic children, matching by gender and age (4–14 years) were enrolled. The diabetic children were divided: a) 20 children in good metabolic control (Hb1ac≤7.5) and b) 48 children in bad metabolic control (Hb1ac>7.5). Dietary and oral hygiene habits were investigated. Caries status was registered using the International Caries Detection and Assessment System. Oral microflora was analysed using the checkerboard DNA-DNA hybridisation method. Plaque acidogenicity was recorded after a sucrose rinse.

**Results:**

Sugared beverage and snack intake was higher in diabetic group compared to non-diabetic group (p = 0.03 and p = 0.04, respectively) and in subjects in bad metabolic control (p = 0.03 and p<0.01, respectively). Oral hygiene habits were similar, except for the use of fluoridated adjuvants, higher in non-diabetic children (p = 0.04). No statistically significant differences were observed regarding caries figures, but a higher number of caries free subjects was found in diabetic subjects in good metabolic control (p<0.01). Significant difference for the main cariogenic bacteria was found between diabetic and non-diabetic subjects (p<0.05). The pH values showed statistically significant differences between diabetic and non-diabetic subjects and between diabetic subjects in good and bad metabolic control (p<0.01).

**Conclusions:**

Diabetic children in good metabolic control might even be considered at low caries risk, while those in bad metabolic control showed an oral environment prone to a high caries risk.

## Introduction

Diabetes mellitus is a chronic disease resulting from a relative or absolute deficiency of insulin, which affects the metabolism of carbohydrate, protein, and fat [1]. Finland, Sardinia (Italy) and Sweden are known to have the highest incidence of Type 1 diabetes in the world [2–6]. Tailored dietary advices are given to children and their families about the amount, type and distribution of carbohydrate to include in main meals and snacks (if appropriate) during the day to promote optimal growth and blood glucose control [7]. Nutrition education and lifestyle counseling should be adapted and delivered both to the individual child and family.

While the association between oral health and Type 2 diabetes is well recognized [8–10] there is still limited evidence available regarding the association between Type 1 diabetes and oral health, even if various oral problems have been reported including an increased presence of caries [11–13].

Dental caries remains one of the most common chronic disease with a dietary-bacterial aetiology [14]. Caries is characterized by an ecological shift within the dental biofilm environment, driven by frequent access to fermentable dietary carbohydrates, leads to a move from a balanced population of microorganisms of low cariogenicity to a microbiological population of high cariogenicity (more aciduric and acidogenic) and to an increased production of organic acids [15]. The acid production near the tooth structures produces the demineralization of enamel and dentin and subsequently may evolve in the development of a cavitation [16].

Unbalanced diabetes is associated with significant cariogenic changes in the oral environment including less resting and stimulated whole saliva, lower saliva buffering capacity and acidic pH, higher salivary glucose, higher salivary albumin concentrations, high proportion of salivary mutans streptococci and yeast [11, 12, 17–19]. Changes in the oral microflora of diabetic subject in poor glycemic control may significantly influence the prevalence of gingivitis and caries [20, 21]. However, contrary to previous findings, lower caries experience was also reported [22, 23]. No significant differences regarding caries experience between Type 1 diabetic and non-diabetic children was described [24], even if the number of untreated dental caries was higher among the diabetic children, reflecting a lower dental attendance. A high caries susceptibility was also reported in children and adolescents with Type 1 diabetes mellitus in poor bad metabolic control [18, 25, 26]. Poor metabolic control is defined as a HbA1c level exceeding the target range of HbA1c for all age-groups of < 7.5% (58 mmol/mol) [7].

From these premises, there is the need to understand the association between Type 1 diabetes and oral health, especially caries, that has still a high prevalence in children populations; this association might be critical for the diabetes long-term management [27]. The number of people affected by diabetes in Sardinia is estimated in almost 95,000 individuals (56 cases/1000 individuals). In Sassari area 1,013 children affected by Type 1 diabetes are reported (41 cases/1000 individuals) [28]. To reply to this need, caries experience and caries-related variables between diabetic and non-diabetic children were compared; a comparison was also performed among the diabetic children according to their metabolic status. The null-hypothesis was that no difference regarding caries experience between diabetic and non-diabetic children and among diabetic children with differences in the metabolic control would be observed.

## Material and methods

### Study design and sample

The study protocol was approved by the Ethical Committee of the University of Sassari, Sassari, Italy [authorisation number 133/2014] and conducted according to the principles of the Helsinki Declaration II. The study was conducted from January 2015 to September 2015. A cross-sectional case control study (rate 1:2 matched for age and gender) was designed. Two categories of subjects were enrolled, diabetic and non-diabetic children (aged 4–14 years old). The criteria necessary for the enrolment into the study were: diabetes diagnosed from more than 2 years [7], within 14 years of age, living in Sassari and surrounding area, good general health apart from diabetes, reporting to clean teeth at least twice a day.

Power analysis was performed before the start of the study using the web-based Openepi^TM^ platform (http://openinfo.com), considering a caries prevalence of about 50% [29]; sample size was increased by 20% bearing in mind a modification in caries prevalence and a high number of non-responders. The number of diabetic children needs to be enrolled was fixed in 64 with an actual power of 0.95. Information about the study program was mailed to 225 parents/guardians of 75 diabetic and 150 non-diabetic children, asking the consent for their child to participate into the study. Seventy-two diabetic children agree to participate and 68 were enrolled, while from the group of 150 non-diabetic children selected, 136 subjects, matching by gender and age were also enrolled. The diabetic children were divided into two subgroups according to data from their medical charts: a) 20 children with a good metabolic control (Hb1ac≤7.5) and b) 48 children with bad metabolic control (Hb1ac>7.5) [30, 31].

### Questionnaire

A limited checklist of 19 foods and beverages (sugar-sweetened beverages, savoury snacks and sweets) with a frequency response section was parents/self-administered to the subjects to report how often each item was consumed over a specified period [32, 33]. The oral hygiene habits were investigated by questions on regular use and frequency of toothbrushing, use of fluoridated toothpaste, use of others fluoridated products and, frequency of dental attendance [34].

### Clinical examination

The clinical examination was made under optimal lighting using a mirror and a World-Health-Organization probe. The WHO probe has a coloured band (called the reference marking) located 3.5–5.5 mm from the probe tip. For caries registration, the International Caries Detection and Assessment System (ICDAS) [35] index was used. No radiographs for caries diagnosis were used [36]. For the diabetic children, data on their medical condition was also retrieved from their medical charts.

### Saliva samples and microbiological analyses

All subjects were instructed not to brush their teeth or to eat/drink during one hour prior to the oral examination.

Saliva sample was collected using paraffin gum during 5 min with continuously spitting into a test tube after 60 sec of pre-stimulation chewing one piece of paraffin [37]. Younger children were instructed on the collection procedure and followed during the test, inviting them repeatedly to spit during the 5 minutes of chewing. The saliva samples were sent to Department of Microbiology, University of Bologna for evaluation of oral microflora.

The microbiological analysis was made using the checkerboard DNA-DNA hybridisation method [38]. Whole genomic probes were matched from 15 bacterial strains (*Streptococcus mutans*, *Streptococcus sobrinus*, *Streptococcus criceti*, *Streptococcus downei*, *Streptococcus ferus*, *Streptococcus macacae*, *Streptococcus ratti*, *Streptococcus infantis*, *Streptococcus mitis*, *Streptococcus gordoni*, *Lactobacillus salivarius*, *Streptococcus sanguinis*, *Streptococcus salivarius*, *Lactobacillus casei*, *Lactobacillus fermentum)* known to be associated with caries. Matching the obtained signals with the ones generated by the pooled standard samples, containing a count of 10^6^ and 10^5^ of each bacterial species, respectively, an evaluation of the bacterial count was performed in the samples.

### Plaque acidogenicity

Immediately after the saliva sampling, the plaque acidogenicity was assessed using the pH indicator strips in the interproximal space in 2 sites: 1) between the first and the second maxillary primary molars right and left in the younger children or 2) between the 2^nd^ primary molar and 1st maxillary molar right and left in the older children. The strips measure a pH value in the range of 4.0–7.0 (Spezialindikator, pH range 4.0–7.0; Merck, Darmstadt, Germany) [39]. Each strip was cut into 4 pieces (approx. 2 mm in width) to get a strip that more easily could be inserted into the interproximal space. The strip was held *in situ* for 10 s after which it was removed and its colour compared to the colour index scheme supplied by the manufacturer. The pH was determined to one decimal of the value. For each site, 3 measurements were carried out. Measurements were performed before (0 min) and at 2, 5, 10, 15, 20 and 30 min after a mouth rinse with 10% sucrose for 1-min.

### Statistical analyses

All the data were input into a spreadsheet (Microsoft Excel® 2011 for Mac, version 14.4.3). Statistical analyses were performed using Stata/SE^®^ software, version 13.1 for Mac (64-bit Intel^®^).

Regarding questionnaire items, subjects were asked to choose from seven options ranging from never (zero intake) to more than four times per day and a score was given to each option (*i*.*e*. never or zero intake = 0, once a week = 1 etc.). The scores were then added for each participant as sugared beverage intake total score and sugared snack intake total score.

Data from the dental clinical examinations were grouped as follows: healthy/caries-free (ICDAS 0), initial (caries in enamel ICDAS 1–2), moderate (caries not cavitated ICDAS 3–4), and severe (cavitated caries in dentin ICDAS 5–6).

Data from microbiological analysis was coded on a scale from 0 to 5: 0 = no signal; 1 = a signal density weaker than that of the low standard (<10^5^ bacteria); 2 = a signal density equal to that of the low standard (= 10^5^ bacteria); 3 = a signal density higher than that of the low standard but lower than that of the high standard (>10^5^ but <10^6^ bacteria); 4 = a signal density equal to that of the high standard (= 10^6^ bacteria) and 5 = a signal density higher than that of the high standard (>10^6^ bacteria).

The mean plaque pH (± Standard Error) for all subjects at the different time points was calculated for the two interproximal sites. The maximum pH fall and minimum pH after the sucrose rinse were calculated for each subject. The pH curve, as the area below the critical pH of enamel (AUC_5.7_) and of dentine (AUC_6.2_) was also calculated [40].

Comparisons of the different variables were made between the diabetic and non-diabetic subjects and between diabetic subjects in good metabolic control and in bad metabolic control. All data was analysed univariately to describe the variables and distributions. Student t test between the two groups was calculated, and p<0.05 was considered as a significant level. To avoid the attenuating effect of unequal variability among groups on the value of t, a square root transformation was performed when the response variable was a count. One-way analysis of variance (ANOVA) was performed for means comparison between diabetic subjects in good metabolic control, in bad metabolic control, and non-diabetic subjects. Moreover, a two-way table analysis (chi-square) was conducted to determine the association between diabetic and non-diabetic subjects and frequency of outcomes from the questionnaire items. If a cell contained a value less than five the Fisher’s exact test was calculated.

## Result

Gender distribution was 33 males and 35 females in the diabetic group and exactly the double in the non-diabetic group; the mean age of the sample was similar in both groups (12.11+2.77 in diabetic group and 12.09+2.68 in non-diabetic group).

### Diet and oral hygiene habits

Sugared beverage and snack scores were significantly statistically higher in diabetic group compared to non-diabetic group (p = 0.03 and p = 0.04, respectively); the scores were even higher in diabetic subjects in bad metabolic control respect to those in good metabolic control (p<0.01 for sugared beverage and p = 0.03 for snacks) (Table 1).

**Table 1:** Sugared snacks and beverages intake in diabetic and non-diabetic children. The replies were treated as continuous ordinal variables.

**Sugared snacks and beverages intake**	**Diabetic**			**Non-diabetic**	**Comparison**			
	*mean±SD*			*mean±SD*	*P value*			
					non-diabetic group *vs*			diabetic
	total	good metabolic	bad metabolic		diabetictotal	bad metabolic	good metabolic	good metabolic *vs* bad
	N = 68	N = 20	N = 48	N = 136				
*Sugared beverages*	4.10±2.66	2.83±1.22	5.23±2.81	2.47±1.24	**0.03**	**<0.01**	**<0.01**	**<0.01**
*Sugared snacks*	4.43±2.61	2.00±1.90	5.68±2.65	2.16±1.35	**0.04**	**0.03**	**0.03**	**0.03**

The replies to the oral hygiene items (Table 2) were similar between diabetic and non-diabetic subjects, except for fluoridated adjuvants (p = 0.04). The comparison between diabetic in good and bad metabolic control was statistically significant for the use of fluoridated toothpaste and frequency of toothbrushing for more than 2 minutes (p = 0.03); moreover, the use of fluoridated toothpaste was also statistically significant different between diabetic subjects in bad metabolic control and non-diabetic subjects (p<0.01).

**Table 2:** Oral hygiene habits and frequency of dental attendance in the examined groups. The association between diabetics and non-diabetics and frequency of outcomes from the items was determined with chi-square test.

**Behavioural variables**	**Diabetic**			**Non-diabetic**	**Comparison**			
	*mean±SD*			*mean±SD*	*P value*			
					non-diabetic group *vs*			diabetic
	total	good metabolic	bad metabolic		Diabetic total	bad metabolic	good metabolic	good metabolic *vs* bad
**Fluoridated toothpaste**								
*No*	21 (38.9)	10 (50.0)	11 (22.9)	61 (44.8)	0.36	**<0.01**	0.72	**0.03**
*Yes*	47 (69.1)	10 (50.0)	37 (77.1)	75 (55.2)				
**Other fluoridated adjuvants**								
*Yes*	8 (11.8)	3 (15.0)	5 (10.4)	15 (11.0)	**0.04**	0.69	0.72	0.83
*Sometimes*	12 (27.6)	6 (30.0)	6 (12.5)	32 (23.5)				
*No*	48 (70.6)	11 (55.0)	37 (77.1)	89 (65.5)				
**Toothbrushing twice/day ≥ 2 min**								
*No*	49 (72.1)	13 (65.0)	36 (75.0)	98 (72.1)	0.93	0.84	**0.02**	**0.03**
*Sometimes*	11 (16.2)	2 (10.0)	9 (18.7)	27 (19.8)				
*Mostly every day*	8 (11.7)	5 (25.0)	3 (6.3)	11 (8.1)				
*Every day*	-—- -	-—- -	-—- -	-—- -				
**Dental attendance**								
*In pain*	15 (22.1)	2 (10.0)	13 (27.1)	33 (24.3)	0.74	0.58	0.08	0.12
*Every six months*	18 (26.5)	4 (20.0)	14 (29.2)	39 (28.6)				
*Once a year*	35 (51.4)	14 (70.0)	21 (43.7)	64 (47.1)				

### Caries data

**Fig 1:**
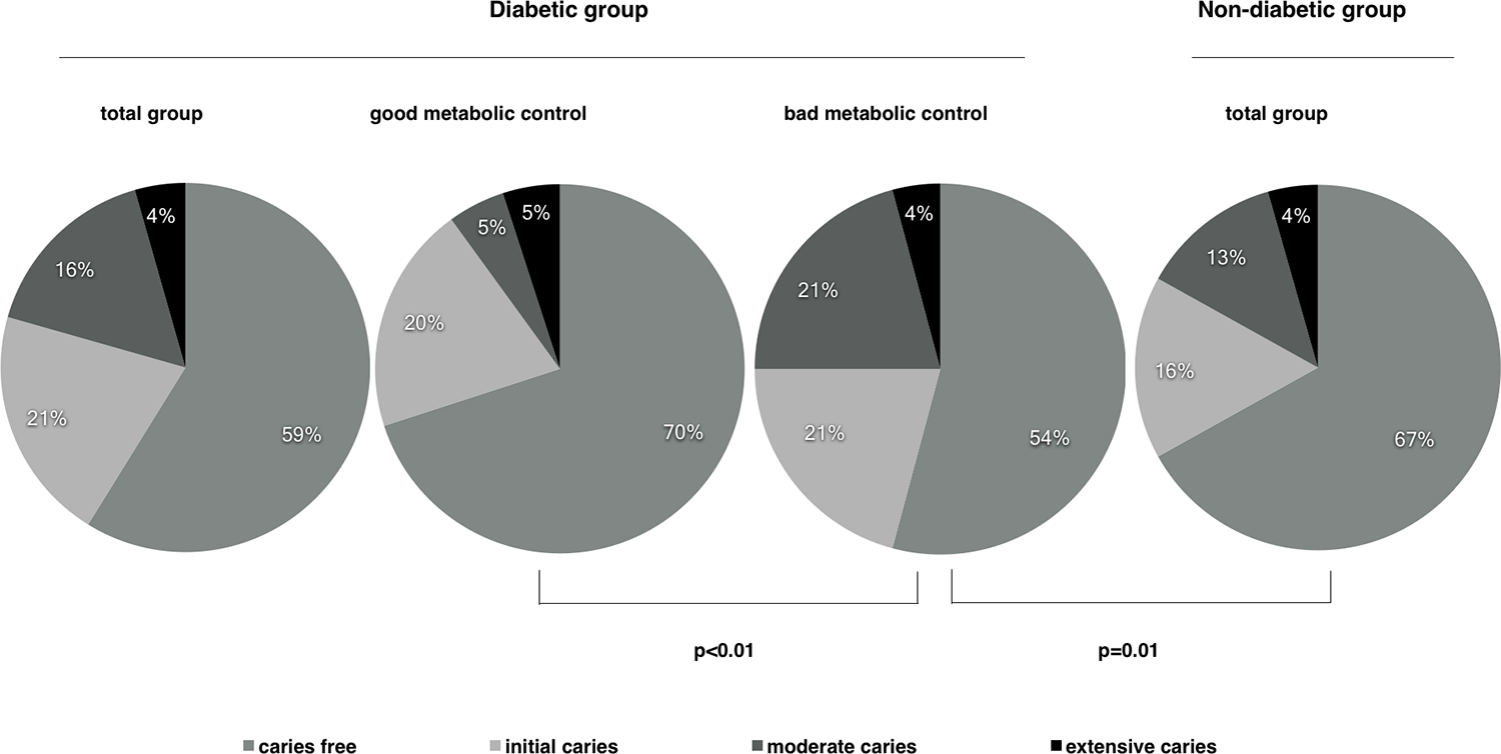
Caries figures in diabetic (good and bad metabolic control) and non-diabetic groups.

Caries free varies from 54% in diabetic subjects in bad metabolic control to 70% in diabetic subjects in good metabolic control (Fig 1). Overall no statistically significant differences were observed between diabetic and non-diabetic groups regarding caries prevalence, while the caries free subjects were statistically significant higher in diabetic group in good metabolic control compared to diabetic group in bad metabolic control (p<0.01). The others caries figures (Initial, Moderate and Extensive) were similar in all groups.

### Microbiological analyses

No difficulties were found in the saliva collection even for the younger children since the sample procedure was proposed as a game. The association between bacteria strains and diabetic and non-diabetic subjects is displayed in Table 3. The bacteria strains were categorized in primary cariogenic bacteria (*S*. *mutans*, *S*. *sobrinus*, others mutans streptococci, *L*. *casei*, *L*. *fermentum*) and not primary cariogenic bacteria (*S*. *sanguinis*, *S*. *salivarius*, *S*. *mitis*, *S*. *gordonii*, *L*. *salivarius*).

**Table 3:** Numbers of salivary bacteria in paraffin-stimulated saliva based on analysis of a 5-unit score (1 = <10^5^, 2 = 10^5^, 3 = 10^5^>x<10^6^, 4 = 10^6^, 5 = >10^6^).

**Bacterial strains**	**Diabetic** *mean±SD*			**Non-diabetic** *mean±SD*	**Comparison** *P value*			
					non-diabetic group *vs*			diabetic
	total	good metabolic	bad metabolic		diabetic total	bad metabolic	good metabolic	good metabolic *vs* bad
**Primary cariogenic bacteria**								
*S*. *mutans*	3.38±1.15	3.35±1.19	3.35±1.15	2.83±1.07	**0.04**	**0.03**	**0.04**	**0.03**
*S*. *sobrinus*	1.88±0.70	1.70±0.47	1.96±0.77	1.27±0.96	**0.02**	**<0.01**	0.34	**0.04**
others mutans streptococci	2.37±1.12	2.35±1.09	2.37±1.14	2.38±1.19	0.12	0.21	0.51	0.63
*L*. *casei*	2.03±0.90	1.85±0.67	2.10±0.97	1.98±1.03	0.96	**0.02**	0.57	**0.04**
*L*. *fermentum*	2.48±0.97	2.25±0.85	2.58±1.01	2.15±1.21	**0.03**	**<0.01**	0.07	**0.03**
**Not primary cariogenic bacteria**								
*S*. *sanguinis*	2.70±1.01	2.70±1.08	2.71±0.99	2.69±1.01	0.62	0.81	0.96	0.51
*S*. *salivarius*	2.81±1.07	2.60±0.94	2.89±1.11	2.46±0.95	**0.03**	**<0.01**	0.07	**0.04**
*S*. *mitis*	2.46±1.01	2.35±1.04	2.54±1.01	2.22±1.04	0.90	0.95	0.68	0.94
*S*. *gordonii*	2.23±0.98	2.10±0.85	2.29±1.03	2.21±0.87	0.68	0.62	0.48	0.50
*L*. *salivarius*	2.48±0.95	2.45±1.10	2.50±0.90	2.27±1.15	**0.03**	**0.02**	0.86	0.92

A significant association for all primary cariogenic bacteria except for *L*. *casei* and others mutans streptococci was found in the comparison between diabetic and non-diabetic subjects (p<0.05). Statistically significant differences were recorded between diabetic subjects in good and bad metabolic control regarding several cariogenic bacteria (p<0.05). Not primary cariogenic strains, showed not statistically significant differences in the two groups. Only *S*. *salivarius* showed statistically significant differences in the comparison respectively between diabetic and non-diabetic subjects (p = 0.03), between subjects in bad metabolic control and non-diabetic children (p<0.01) and between diabetic subjects in good and bad metabolic control (p = 0.04).

### Plaque pH measurements

**Fig 2:**
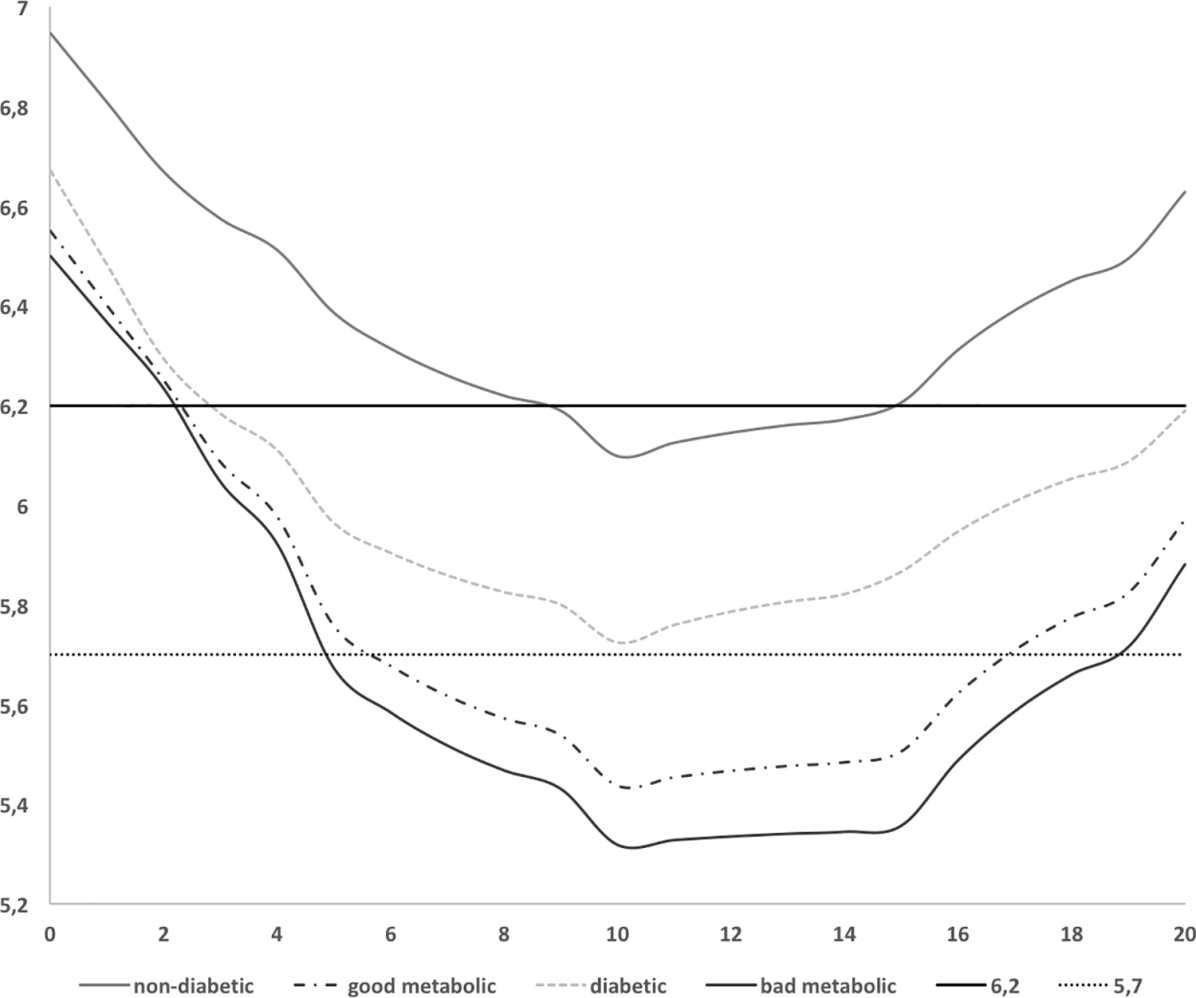
The pH curves at AUC6.2 and AUC5.7 in diabetic (good and bad metabolic control) and non-diabetic groups.

The pH values (Fig 2) showed statistically significant differences for AUC_6.2_ and AUC_5.7_ between diabetic and non-diabetic subjects, between diabetic subjects in good metabolic control and bad metabolic control and between diabetic in bad metabolic control and non-diabetic subjects (p<0.01). The comparison between diabetic subjects in good metabolic control and non-diabetic subjects was statistically significant different (p<0.01) for all pH values except for maximum pH fall (*data not tables)*.

## Discussion

The present study was carried out in order to evaluate the caries figure and the different caries-related variables between Type 1 diabetic and non-diabetic children and among diabetic children according to their metabolic status.

The main outcome was that the oral environment in diabetic population was more prone to caries compared to non-diabetic population even if the caries figures were not statistically significant different, data consistent with the results of previous studies [41]. As in non-diabetic population, in diabetic group a skewed caries distribution was evident. On the other hand, several authors [11, 42] reported a lower prevalence of caries in diabetic group than in non-diabetic population. In this study, a statistically significant higher caries figures were observed in diabetic subjects in bad metabolic control compare to those in good metabolic control. This finding might be linked to several factors like as a higher sugared snack and beverage intake, a non-optimal fluoride exposure, a more cariogenic microbial flora and lower plaque pH values.

A higher intake of sugared snacks and beverages was recorded in the diabetic children compared to non-diabetic; this difference was indeed mostly related to diabetic group in bad metabolic control, since diabetic subjects in good metabolic control reported a sugared foods intake even lower than non-diabetic subjects. International clinical guidelines on the management of Type 1 diabetes call for a healthy diet [43]. The glycemic control may be improved by increasing the intake of foods with low glycemic index [44]. Low carbohydrate (30%-40% energy rate) and very low carbohydrate diets (21–70 g/d) are recommended for the management of Type 1 and Type 2 diabetes, respectively in order to control glycemic values [45]. The higher intake of sugared foods recorded in a sub-group of diabetic children might be considered a risk factor related to the bad glycemic control and the higher caries figures recorded.

Although the regular use of fluoride toothpaste is considered a cornerstone in dental health, producing the dramatic decline of the caries prevalence during the last decades of the 20th century in western countries [46], it might be not sufficient to prevent caries lesions in high risk subjects. In this study, no statistically significant differences were recorded about the use of fluoride toothpaste between diabetic and non-diabetic children; nevertheless considering others fluoridated adjuvants *i*.*e*. fluoride mouthrinse and tablets, a statistically significant difference was found, since diabetic children reported a less frequent use of fluoridated preventive products, contributing to explain the worse clinical situation. In addition, although all children declared to brush their teeth twice a day, the reported duration of the brushing considered effective for a good dental hygiene (≥ 2 minutes) was statistically significant higher in diabetic group in good metabolic control compared to diabetic in bad metabolic control and non-diabetic group. This finding might also contribute to explain the caries figure in the studied populations [47].

Modern concepts regard caries as an interaction among biological, social, behavioural and psychological factors with the dental biofilm as the key element [16]. If a diet rich in fermentable carbohydrates is maintained, prolonged acidic conditions on the tooth surface become frequent and more aciduric bacteria become dominant through acid-induced selection [48]. Early acquisition of mutans streptococci is considered one of the key elements in the development of Early Childhood Caries and a predictable factor of future caries. Another important caries-associated microorganism is *Lactobacillus* species, which colonised carious lesions later than mutans streptococci [49]. Otherwise, the presence in the dental biofilm of some bacterial species as *S*. *sanguinis*, *S*. *salivarius*, *S*. *mitis* and others *Streptoccus spp*. as well as *Lactobacillus spp*. may moderate caries lesion development in children [50]. In the present study different strains of *Streptococcus* and *Lactobacillus spp*. were investigated. Regarding cariogenic bacteria the findings show a higher prevalence of caries-associated pathogens (*S*. *mutans* and *S*. *sobrinus*) in diabetic children compared to non-diabetic children and this difference increased when diabetic children in bad metabolic control were compared to non-diabetic children. In a recent paper, accordingly to our results, mutans streptococci were considered a significant variable affecting caries experience in diabetic children [19]. No differences in the distribution or number of mutans streptococci between Type 1 diabetic and non-diabetic children were also recorded [51]. In the same study significantly lower levels of lactobacilli were found among the diabetic children. This finding is in agreement with our results since *L*. *casei* and *L*. *fermentum* showed statistically significant lower concentrations in diabetic than in non-diabetic children. This finding is probably related to the higher intake of sugared beverages and snacks reported by diabetic children in bad metabolic control.

Stephan-curve was also recorded after a sugar challenge and minimum pH, maximum pH fall, and AUC_6.2_ and AUC_5.7_ were evaluated. Since they were firstly described, pH parameters have been frequently used for the evaluation of food cariogenicity and⁄or individual caries risk status [52]. Findings showed a more acidogenic environment in diabetic subjects compared to non-diabetic group. Differences were also recorded between diabetic children in bad and good metabolic control: all pH parameters were more prone to caries risk in children in bad metabolic control than in good.

Despite the study limitations, primarily the subjects sampled from a limited geographical area, a longitudinal clinical evaluation of the study participants might led to an increase of information on the oral health status of the diabetic young patients.

Both diabetes and caries are complex multifactorial diseases. These results underline that, even if it is difficult to separate specific effects of diabetes-induced changes on the caries process, caries and diabetes recognize diet as a main risk factor in the disease management.

Based on this study’s results, the following conclusions can be summarized:

Diabetic subjects in good metabolic control might even be considered at low caries risk, since they show an oral environment less prone to caries risk compared to non-diabetic population.

Otherwise, diabetic subjects in bad metabolic control showed a worse oral environment and are at high risk of caries.
